# Clinical features of puff adder envenoming: case series of *Bitis arietans* snakebites in Kenya and a scoping review of the literature

**DOI:** 10.1371/journal.pntd.0012845

**Published:** 2025-02-10

**Authors:** Frank-Leonel Tianyi, Cecilia Ngari, Mark Wilkinson, Stanley Parkurito, Elizabeth Chebet, Evans Mumo, Anna Trelfa, Denis Otundo, Edouard Crittenden, Geoffrey Maranga Kephah, Robert A. Harrison, Ymkje Stienstra, Nicholas R. Casewell, David G. Lalloo, George O. Oluoch

**Affiliations:** 1 Department of Tropical Disease Biology, Centre for Snakebite Research and Interventions, Liverpool School of Tropical Medicine, Pembroke, United Kingdom,; 2 Department of Clinical Sciences, Liverpool School of Tropical Medicine, Pembroke, United Kingdom,; 3 Kenya Snakebite Research and Intervention Centre, Kenya Institute of Primate Research, Ministry of Health, Nairobi, Kenya,; 4 Mwingi sub-county hospital, Kitui County, Kenya,; 5 Chemolingot sub-county hospital, Baringo County, Kenya,; 6 Department of Internal Medicine/Infectious Diseases, University of Groningen, University Medical Centre Groningen, Groningen, The Netherlands; College of Health Sciences, Bayero University Kano, NIGERIA

## Abstract

**Introduction:**

The puff adder (*Bitis arietans*) is a medically important snake species found across much of Africa, yet there is limited literature on the clinical features and pathophysiology of envenoming after a puff adder bite.

**Methods:**

We conducted a case-series study to describe the clinical features of patients with puff adder bites who were treated in two primary healthcare facilities in Kenya and complemented our case-series with a scoping review of all published cases of puff adder envenoming that contained sufficient clinical details to highlight the major features.

**Results:**

Between December 2020 and September 2021, 15 patients were admitted with a suspected puff adder bite (based on the patient’s description of the biting snake or confirmed in patients who brought the dead snake or a picture of the biting snake for identification) at the Chemolingot and Mwingi sub-county hospitals in Baringo and Kitui counties, central Kenya. Common local and systemic features on admission included pain (n=15, 100%), swelling (n=14, 93%), and haemorrhage (n=9, 60%). Coagulopathy (n=2, 13%), blistering (n=1, 8%) and shock (n=1, 8%) were less common. In addition, we conducted a literature review and identified 23 studies with detailed descriptions of the clinical features of puff adder envenoming from 37 patients. Local features were common and consistent across cases—swelling (100%, n=37) and pain (95%, n=35). Systemic features were less consistent, with 10 (27%) patients exhibiting hypotension on admission, 10 (27%) patients reporting a fever, and 13 (35%) developing anaemia. Some complications were more common in patients with bites by captive snakes (amputations), compared to patients with bites by wild snakes (hypotension). Snake identification was easier and more accurate after bites by captive snakes, but more challenging for patients bitten in community settings.

**Conclusion:**

We combined clinical cases and a literature review to describe the common and less common clinical features of puff adder envenoming. Further clinical research incorporating serial laboratory assays of patients with definitively identified puff adder bites is crucial to better understand the pathophysiology of envenoming by this medically important snake species.

## Author summary

The puff adder is a medically important snake species found across much of Africa, yet the signs and symptoms of patients presenting with a puff adder bite have not been extensively described. We described the signs and symptoms of patients who were admitted with a puff adder bite in two primary healthcare facilities in Kenya and identified further published cases from the literature with sufficient details of the clinical presentation. Between December 2020 and September 2021, of the 15 patients admitted with a diagnosis of a puff adder bite at the Chemolingot and Mwingi sub-county hospitals, pain, swelling and haemorrhage were most common, while coagulopathy, blistering and shock were less common. From the 23 studies which described signs and symptoms from 37 patients, swelling and pain were most common, while hypotension, fever, and anaemia were less common. It was easier to objectively identify the biting snake after bites by a captive snake than after a bite in the community, and complications like amputations and hypotension followed the same pattern. Our study describes the common and less common clinical features of puff adder envenoming, but further clinical research incorporating serial laboratory assays of patients with definitively identified puff adder bites is crucial to better understand the pathophysiology of envenoming by this medically important snake species.

## Introduction

The puff adder (*Bitis arietans*) is one of the most medically important snakes in the world. It is a large terrestrial snake that hunts by ambush, has a broad geographic range (occurs naturally in 46 African and Middle Eastern countries), and has a remarkable ability for camouflage [1]. It is commonly accidentally stepped upon by an unsuspecting victim in the savannah regions of sub-Saharan African countries, resulting in frequent bites to the lower limbs [2]. It is a popular attraction in zoos and among exotic pet traders. This expands the risk of a snakebite beyond the African continent, to Europe, North America, and Asia, where these snakes do not occur naturally.

Despite a high anecdotal burden of bites, there has been little empirical evidence detailing the epidemiological burden, or the clinical features of puff adder envenoming. Clinical studies have identified high proportions of puff adder bites among snakebite victims, for example, accounting for up to 75% of snakebites in Zimbabwe [3]. This study did not describe the clinical features of puff adder victims separately, precluding a deep understanding of the pathophysiology of puff adder envenoming. In 1975, Warrell et al [4] described the clinical details of 10 patients bitten by puff adders in the North of Nigeria, and almost 50 years later, this remains the most detailed study of puff adder envenoming in the academic literature. For an old-world viper with widespread geographic coverage, this is concerning because this single viewpoint on the features of puff adder envenoming may limit the recognition of puff adder envenoming in other regions, delay effective antivenom treatment, and increase the risks of adverse outcomes. This problem is accentuated by the heterogeneity in venom composition from puff adders within and between countries and regions [5]. The clinical, functional, and therapeutic consequences of other medically important snake species have been investigated and described [6–8], but not so for puff adders. This can be partly explained by the limited clinical research infrastructure in the primary health facilities that receive the most patients with puff adder bites, and the limited funding to support snakebite research [9,10].

In this study, we aim to provide a comprehensive description of the clinical features of puff adder envenoming in patients treated in two primary healthcare facilities in Kenya and complement our case-series with a review of all published cases of puff adder envenoming that contained sufficient clinical details to highlight the major features of puff adder envenoming.

## Methods

### Ethics statement

Ethical approval for the case series portion of this study was obtained from the Kenyatta National Hospital—University of Nairobi and Ethics Research Committee, Nairobi, Kenya (P149/03/2020) and the Liverpool School of Tropical Medicine Research Ethics Committee (Research Protocol 18–058). Written informed consent was obtained from the participants prior to data collection. For participants less than 18 years of age, their assent was sought directly, and written consent was obtained from their legal guardians. A witnessed consent process was used for patients who could not read or write.

### Case series

A clinical observational study was conducted in Baringo and Kitui Counties in Kenya, as part of the NIHR funded African Snakebite Research Group project. All patients who presented to the Chemolingot sub-county hospital (Baringo County) and the Mwingi sub-county hospital (Kitui County) with a suspected puff adder bite between 01 December 2020 and 30 September 2021 were reviewed by the study clinician who has expertise in managing puff adder envenoming. We included patients who presented within 24 hours of the bite and were ≥8 years old. Snake identification was by either visual inspection of the dead biting snake, a picture of the biting snake, or a detailed description of the snake to allow its identification by the treating team.

### Clinical evaluation

Case report forms were used to collect data on patient demographics, bite circumstances, bite characteristics, time of bite and time of hospital arrival, 20-minute whole blood clotting test (20WBCT) results, and details of antivenom treatment (other clinical indications, administration times, brand, dosage, adverse reactions).

### Statistical analyses

Descriptive analysis of sociodemographic, bite circumstances and clinical data was carried out using proportions, means, and standard errors (SEs) for normally distributed variables and medians and interquartile ranges (IQRs) for nonparametric variables. Symptoms and signs were grouped into local and systemic envenoming and figures were created displaying the frequencies of each. Stata v16.1 was used for statistical analyses and a p-value <0.05 was used for statistical significance.

### Review

A single author (FLT) searched the PubMed database using the keywords (“puff adder”) OR (“*Bitis arietans*”), and the Global Index Medicus database using the keywords (tw:(puff adder)) OR (tw:(*Bitis arietans*)) on 15^th^ July 2023. All references, with no language restriction, were imported into EndNote version 20, duplicates were removed, and the references exported to Covidence systematic review software for screening and selection. Titles and abstracts were reviewed, and we included studies that involved human snakebite patients with no age restriction and described a clinical case or a series of cases of puff adder envenoming. Only clinical cases with information available on the snake species (confirmed or suspected), bite location, and local and/or systemic symptoms and outcome were included. We excluded animal studies, in-vivo assays or observational studies in which symptoms and signs of envenoming were aggregated, preventing a detailed description of individual cases. Extracted data included last name of first author, year of publication, country where study was conducted, demographic data, salient clinical features, treatment, and outcome. Where available, detailed laboratory data were extracted and summarised as appropriate. We extended our search to include the reference lists of included articles, google scholar, and conference proceedings. The PRISMA-Scoping Review guidelines were used in the conduct and reporting of the review section of this paper and a checklist is available in the supporting information section (S1 Checklist) [11].

### Inclusivity in global research

Additional information regarding the ethical, cultural, and scientific considerations specific to inclusivity in global research is included in the Supporting Information (S2 Checklist).

## Results

### Clinical study

Between December 2020 and September 2021, 15 patients were admitted with a puff adder bite at the Chemolingot (53%, n=8) and Mwingi (47%, n=7) sub-county hospitals and enrolled into our study.

### Study population

The median age of study participants was 23.0 years (IQR: 16.0–35.0), and 53% were male as presented in Table 1. Patients had a median delay to hospital of 8.1 hours (IQR: 5.3–11.3) after the snakebite. Herding was the most common occupation among patients (27%, n=4) and only two (13%) patients attended a traditional healer before hospital presentation. Most patients were walking outdoors when they were bitten (47%, n=7), though some were sleeping (33%, n=5), mostly outdoors, when they were bitten. The lower limbs were the most commonly bitten body part (73%, n=11).

**Table 1.** Characteristic of puff adder patients in Kenyan clinical case series.

Patient characteristics	Total (N=15)
Age, median (IQR)	23.0 (16.0–35.0)
Gender	
Male	8 (53%)
Female	7 (47%)
Occupation	
Herder	4 (27%)
Students	1 (7%)
Housewife	2 (13%)
Unemployed	3 (20%)
Unknown	5 (33%)
Activity when bitten	
Outdoors/Walking	7 (47%)
Toilet visit	1 (7%)
Sleeping	5 (33%)
Indoors	2 (13%)
Bite location	
Foot	6 (40%)
Ankle/knee	5 (33%)
Hand/Forearm	2 (13%)
Arm/Shoulder	1 (7%)
Face	1 (7%)
Visited a traditional healer	
Yes	2 (13%)

### Biting snake identification

**Fig 1:**
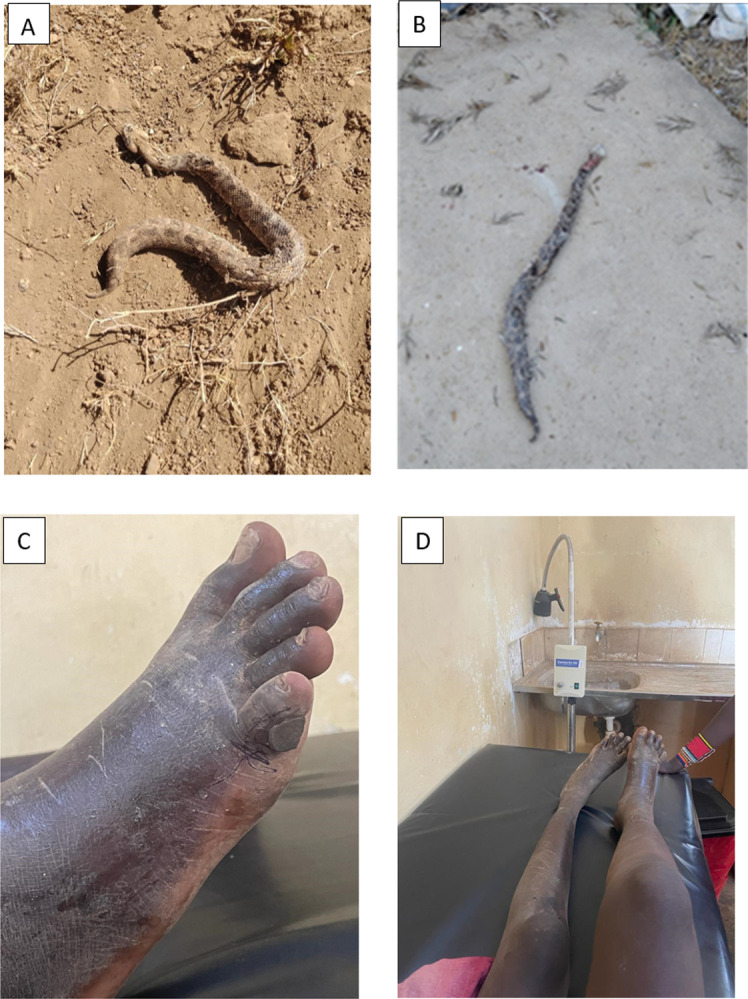
Biting snake and local effects of bite. A*. Picture of biting snake taken by snakebite victim and used for snake identification. B*. Picture of biting snake brought to the health facility for snake identification. C. Puff adder bite to the right small toe, and black stone applied as an early intervention (Patient bitten by snake in Fig 1B above). D. Progressive swelling of entire right leg after a puff adder bite to the right small toe (Patient bitten by snake in Fig 1B above). *Both snakes identified as *Bitis arietans* by LSTM herpetologist Edouard Crittenden and KSRIC herpetologist Geoffrey Kephah.

A combination of objective and subjective measures were used to identify the biting snake species. The biting snake was killed by the victim in three cases. One of the patients brought the dead snake to the hospital for identification, one patient took a picture of the dead snake and shared this with the healthcare workers, and the last patient provided a description of the dead snake and recognised the snake on a picture chart of medically important snakes in Kenya. Fig 1 shows pictures of the puff adders, and the bitten limb of one of the patients. The remaining 12 patients saw the biting snake, but the snake was not killed and they did not take a picture of the biting snake. The identification was made following a description of the biting snake, and a recognition of the snake on a picture chart of medically important snakes in Kenya. Despite being less objective, the latter identification method is worth considering because puff adders are very common in Kenya, they make a distinctive sound (puffing air from their body) before biting, and they are known locally as “*Kipsu*” in Baringo county and *“Kimbuva”* in Kitui county.

### Features of local and systemic envenoming

All 15 patients complained of pain on admission which was very severe in five (33%). There was an improvement in pain over time; at 24 hours post admission, no-one had persisting very severe pain, only one (7%) patient reported severe pain, and seven patients reported decreases in the level of pain. The most common local sign was swelling of the bitten part, present in 14 (93%) patients on admission, persisting through 12 hours in all, and still present in 12 (80%) at 24 hours post admission. Fang marks were visible in ten patients (67%) on admission, while six patients (40%) patients had bruises, and one patient had a blister around the bite site. Bruises were more commonly detectable 6 hours after admission, with 10 (67%) patients having bruises at this time point.

Haemorrhage was the most common feature of systemic envenoming on admission, present in nine (60%) patients, occurring in patients both from Chemolingot and Mwingi. The sites of haemorrhage were varied and included the bite site (n=8, 53%), gums (n=2, 13%) and venepuncture site (n=2, 13%), with some patients bleeding from more than one site. At 6 hours, four (27%) patients still had signs of haemorrhage, with three (20%) bleeding from the bite site, and two (13%) bleeding from the gums. Coagulopathy, evidenced by an abnormal 20WBCT, was present in only two (13%) of patients on admission and one (7%) patient 24 hours later, and these patients were all from Mwingi sub-county hospital. Shock, defined as a systolic blood pressure less than 90 mmHg, was present in one (6%) patient on admission, and the low BP persisted at 6 hours, and resolved at 24 hours.

### Treatment and outcomes

All patients were treated with Inoserp Pan-Africa antivenom (500 LD_50_; a polyvalent anti- *Bitis* spp., *Dendroaspis* spp., *Echis* spp., and *Naja* spp. specific equine F(ab)’_2_ antivenom manufactured by Inosan Biopharma, Spain). Antivenom treatment indications were: rapid progressive swelling (RPS) extending to the fingers or toes in three patients, swelling involving more than two joints in two patients, swelling involving half of the limb in nine patients, and one patient having spontaneous bleeding [12]. Most patients had more than one feature of envenoming, but only one was recorded as the indication for antivenom (for example, the indication for two patients with an abnormal 20WBCT on admission were RPS and spontaneous bleeding). No patients required surgical intervention, and there were no fatal outcomes. Details of the clinical features are presented in S1 Table.

### Literature review

**Fig 2:**
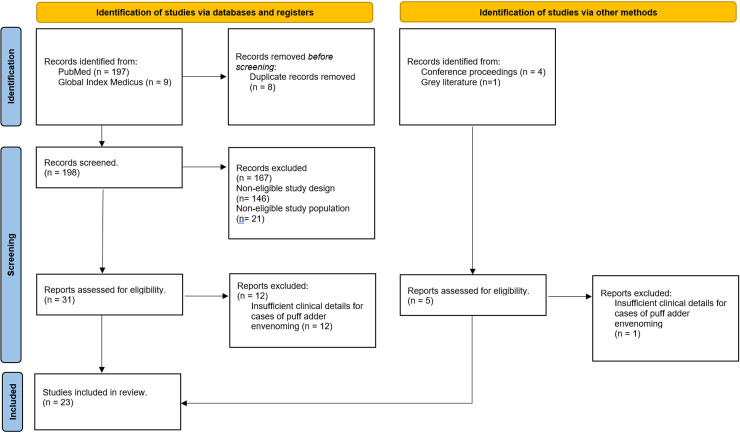
PRISMA flow chart of studies assessed for this review.

Our literature search returned 206 articles, of which eight were duplicates. After screening the titles and abstracts, 31 articles met our criteria for full text review, and 12 were excluded. We identified five further articles from our extended search of the reference lists of included articles, conference proceedings, and a cursory search of google scholar. We extracted and summarised data on 37 cases of puff adder bites, published in 23 studies between 1970 and 2023, nine (39%) studies were in sub-Saharan Africa [4,13–19], six (26%) in North America [21–26], six (26%) in Europe [27–32], and two (9%) in Asia [33,34]. The median age of all patients was 32.5 years (range 1–64), and 75% of patients were male. Bites were on the hands or fingers in 77% of cases, and this was most common among snake handlers in non-African countries. Fig 2 shows the PRISMA flow chart on studies in this review, while Table 2 presents a summary of sociodemographic and clinical features from the included articles.

**Table 2:** Summary of sociodemographic and clinical features of published cases of puff adder envenoming.

No	Author	Year	Location	Number of cases	Age (yrs)/ Median age(range)	Gender	Captive/Wild snake	Bite sites	Clinical signs and symptoms	Antivenom name (%)	Surgical care/ %	Outcome
1	Takahashi et al [26]	1970	USA	1	30	Male	Captive	Right index finger	Fang marks Local bleeding Severe pain Progressive swelling Swollen tender axillary lymph nodes Fever	SAIMR polyvalent (100%)	Nil	Full recovery
2	Sezi et al [19]	1972	Uganda	2	13 50	Male Male	Wild	Right foot (50%) Left hand (50%)	Fang marks (50%) Severe swelling (100%) Hypotension (100%) Anaemia (100%) Local bleeding (100%) Distal haematoma (50%) Fever (50%)	Polyvalent anti-snake serum (100%)	Surgical debride-ment and skin grafting (50%)	Full recovery (100%)
3	Warrell et al [4]	1975	Nigeria	10	24.5 (9–55)	NR	Wild	Calf (40%) Ankle (20%) Heel (10%) Toe (10%) Hand (10%) Thumb (10%)	Fang marks (40%) Local bleeding Bleeding gums (10%) Epistaxis (20%) Hypotension (20%) Severe pain (100%) Progressive swelling (70%) Blisters (50%) Fever (50%) Vomiting (30%) Tender lymph nodes (10%) Jaundice (20%) Anaemia (30%)	SAIMR polyvalent (70%)	NR	Deaths (20%)
4	Reid [28]	1978	UK	5		NR	Captive	Left middle and index fingers (20%) Left hand (20%) NR (60%)	Severe pain Progressive swelling (20%) Systemic poisoning (40%) Mild poisoning (20%)	Antivenom name not given (80%)	NR	Necrosis (60%)
5	Sigel et al [29]	1980	Germany	1	29	Male	Captive	Right thumb	Progressive swelling Local bleeding Blisters Signs of compartment syndrome	Polyvalent antivenom	Fasciotomy of the forearm	Full recovery
6	Schweitzer et al [18]	1981	South Africa	1	16	Female	Wild	Left index finger Right hand	Fang marks Local bleeding Severe pain Progressive swelling Clinical diagnosis of compartment syndrome in left forearm Bilateral carpal tunnel syndrome	SAIMR polyvalent (100%)	Bilateral carpal tunnel release Fasciotomy of the forearm	Full recovery
7	Brossy et al [14]	1982	South Africa	1	NR	Male	NR	Left index finger	Fang marks Progressive swelling	Polyvalent antivenom	Nil	Full recovery
8	Hamby et al [24]	1983	USA	1	29	Male	Captive	Right hand (thenar region)	Fang marks Severe pain Progressive swelling	Antivenom name not given	Nil	Full recovery
9	Theakston et al [31]	1985	UK	1	21	Male	Captive	Left fifth finger	Severe pain Progressive swelling Tender swollen axillary lymph nodes Fever	No antivenom	Nil	Full recovery
10	Pugh et al [17]	1987	Nigeria	2	35 (20–50)	Female	Wild	Toe (50%) Foot (50%)	Severe pain Progressive swelling (50%)	SAIMR polyvalent (50%)	Nil	Full recovery
11	Bey et al [21]	1997	USA	1	35	Male	Captive	Right index finger	Fang marks Local bleeding Severe pain Progressive swelling Ecchymosis	Behringwerke polyvalent (100%)	Debridement	Cutaneous scarring Complete loss fingernail
12	Lavonas et al [25]	2002	USA	1	37	Female	Captive	Right ring finger	Haemorrhagic vesicles Hypotension Ecchymosis Severe pain Progressive swelling Local bleeding Necrotic finger Fever Anaemia	SAIMR polyvalent (100%)	Digit amputation	Digit amputation
13	Le Dantec et al [16]	2004	Senegal	1	24	Male	Wild	Right calf	Severe pain Progressive swelling Local bleeding Hypotension Bleeding from phlebotomy sites Blisters Bullae Necrosis	Fav-Afrique polyvalent	Debridement	Full recovery
14	Strubel et al [30]	2008	Germany	1	20	Male	Captive	Right index finger	Severe pain Progressive swelling Clinical diagnosis of compartment syndrome in the right forearm Signs of impaired coagulation Necrosis	Antivenom name not given	Debridement Fasciotomy Digit amputation	Loss of finger
15	Disser et al [22]	2010	USA	1	43	Male	Captive	Right index finger	Fang marks Severe pain Progressive swelling Finger necrosis	SAIMR polyvalent	Digit amputation	Loss of finger
16	Rainer et al [27]	2010	Austria	1	26	Male	Captive	Right hand	Fang marks (4) Severe pain Progressive swelling Erythema Hypotension	SAIMR polyvalent	Nil	Full recovery
17	Dymarsky et al [23]	2015	USA	1	50	Female	Captive	Neck (left submandibular region)	Severe pain Progressive swelling Anaemia	SAIMR polyvalent	NR	Full recovery
18	Firth et al [15]	2016	South Africa	1	1	Male	NR	Left upper limb	Severe pain Progressive swelling Clinical diagnosis of compartment syndrome Coagulopathy + Anaemia	SAIMR polyvalent	Fasciotomy of the arm and forearm Debride-ment	Death
19	Benjamin et al [13]	2020	Guinea	1	64	Male	Captive	Left index finger	Severe pain Progressive swelling Local bleeding Blisters Coagulopathy	Inoserp Pan-Africa polyvalent antivenom 2 vials Pt declined further vials	Nil	Full recovery
20	Engelbrecht [32]	2021	Ireland	1	22	Male	Captive	Right hand (first web space)	Severe pain Progressive swelling	SAIMR polyvalent	Nil	Full recovery
21	Wakasugi et al [34]	2021	Japan	1	23	Male	Captive	Left middle finger	Severe pain Progressive swelling Low blood pressure Subcutaneous haemorrhage in left arm and back	Nil	Nil	Mild contractures of the middle and ring fingers
22	Husain et al [33]	2023	Malaysia	1	Mid-30s	Male	Captive	Dorsum of both hands	Fang marks Severe pain Progressive swelling Hypotension Coagulopathy Anaemia Distal subcutaneous haemorrhage	Nil	Nil	Death
23	Smit et al [20]	2023	South Africa	1	27	Male	NR	Left wrist	Fang marks Severe pain Progressive swelling Clinical diagnosis of compartment syndrome	Polyvalent antivenom	Nil	Full recovery

### Biting snake identification

The methods of snake identification reported in the literature differed for patients between and within studies. The most accurate and direct methods were by expert identification of exotic snakes that bit their owner (n=12, 32%) [21–24,26–28,30–34], patients that brought the dead snake to the hospital (n=9, 24%) [4,18] and snakes being handled in professional settings (n=7, 18%) [13,19,25,28]. *Bitis arietans* venom was detected in serum samples of patients using ELISA techniques for four patients (11%) [4,17]. For two patients (5%) [16,19], the biting snake was identified based on the patient’s description, and for three patients (8%) the method of identification was not reported [14,15,20]. Overall, 32 (86%) patients had an objectively confirmed puff adder bite, a majority of which were bites by captive snakes, while five (14%) cases were subjectively identified, all of which occurred on the African continent, most likely from bites by wild snakes

### Clinical features of puff adder envenoming reported in the literature

Severe swelling and pain were the most commonly reported local features of puff adder envenoming in the literature, respectively reported by 37 (100%) [4,13–34] and 35 (95%) [4,13,15–18,20–28,30–34] patients. Common local features included fang marks and local bleeding, which were reported respectively by 22 (69%) [4,14,18–22,24,26,27,33] and 23 (62%) [4,13,16,18,19,21,25,26,29] cases. Blisters, necrosis, and ecchymosis were uncommon local features, occurring in eight (22%) [4,13,16,29], six (16%) [4,13,16,29] and four (11%)[21,25] cases respectively. Three patients [4,26,31] developed a tender swelling of the lymph nodes proximal to the bitten body part. One developed a tender swelling in the right axillary and right inguinal lymph nodes after a bite to the right hand [4]. In six (16%) [15,16,18,20,29,30] cases, investigators suspected a compartment syndrome and four [15,18,29,30] of these underwent a fasciotomy.

Systemic features were less consistent across the reported studies. Ten (27%) patients across seven studies [4,16,19,25,27,33,34] had hypotension on admission, with blood pressures as low as 60/30 mmHg [19]. The hypotension resolved after intravenous (IV) fluids combined with antivenom treatment, but it persisted in the two patients who received only IV fluids without antivenom, with fatal outcomes [4,33]. Ten (27%) patients across six studies [4,15,17,19,25,29] reported a fever, with temperature measurements as high as 40.5 °C, and some patients remaining febrile up to 8 days after the bite. Eight of these patients [4,17,19,29] were febrile on admission, while two patients [15,25] developed a fever later during their hospitalisation. Four of these patients [4,15,25] developed local necrosis at the bite site, but there was not enough detail in the articles to confirm or exclude an infected bite wound as the cause of the fever. Thirteen (35%) patients across nine studies [4,16,17,19,23,25,30,33,34] had clinical or laboratory evidence of anaemia, and some had haemoglobin concentrations as low as 5.1 g/dl [19] on arrival. In some cases, the anaemic patients had a normal 20WBCT [4]. All 13 patients had local bleeding from the bite site, and in one patient, the bleeding persisted for three days after the bite. This patient had been admitted and treated with antivenom two days after the bite, and the local bleeding stopped within 24 hours of antivenom treatment [19]. Two patients, who received appropriate antivenom within six hours of the puff adder bite, developed haemorrhagic vesicles close to the bite site, and had an acute drop in haemoglobin concentration (14.3 -> 7.1g/dl [25], and 10.0 -> 7.4g/dl [4]) within eight and 35 hours of the puff adder bite respectively. Two patients had a haemoglobin drop with no external evidence of bleeding (13.4 -> 9.5g/dl [23] and 14.2 -> 9.7g/dl [34]) within 48 hrs of the bite. Some patients developed spontaneous haemorrhage at sites other than the bite wound. The spontaneous haemorrhage was sometimes obvious; for example bleeding gums and epistaxis after a bite to the calf [4]) but could be concealed (a hematoma in the jaw after a bite to the foot [19], and two cases of subcutaneous haemorrhage in the back following bites to the hand [33,34]). One case of spontaneous haemorrhage into the aortic adventitia was detected after an autopsy of a fatal puff adder bite [4]. Five (14%) patients [13,15,16,30,33] had signs of coagulopathy, an abnormal 20WBCT in four cases [13,15,30,33], and prolonged bleeding from a phlebotomy site in one case [16]. Seven (19%) patients [4,16,17,19,33] were reported to have received a transfusion of blood products (whole blood or packed red blood cells) due to acute anaemia after a puff adder bite.

### Treatment and outcomes reported in the literature

Antivenom use varied considerably within and between studies. Five (14%) patients across three studies did not receive antivenom, despite other patients in these same studies receiving antivenom [4,17,28]. Four of these patients were reported to have no indication for antivenom use, and for one patient there was no antivenom in the health facility. In three other cases, no antivenom use was reported at all [31,33,34]: in one case the patient only had mild local signs of envenoming [31], in the two remaining cases, the clinicians were unable to access antivenom [33,34]. Overall, eight (28%) patients did not receive antivenom, with two of them having a fatal outcome [4,17,28,31,33,34]. South African Institute for Medical Research (SAIMR) antivenom (a polyvalent anti *Bitis* spp., *Dendroaspis* spp., *Hemachatus* spp. and *Naja* spp. specific fragmented equine immunoglobulin [F(ab)’_2_] based antivenom manufactured by South African Vaccine Producers [SAVP], South Africa) was administered to 11 (30%) patients [4,17,18,22,23,25,26,28,32], with doses ranging from 20–80 ml. Two patients had an immediate adverse reaction, characterised by wheezing and hypotension [17,25]. The occurrence of late serum reactions was not reported. Schlangengift-Immunserum antivenom (a discontinued polyvalent anti *Bitis* spp., *Echis* spp., and *Naja* spp. equine antivenom, previously manufactured by Behringwerke, Germany) was administered to eight patients [4,19,21]. Inoserp Pan-Africa (500 LD50) [13], Fav-Afrique (a polyvalent anti *Bitis* spp., *Dendroaspis* spp., *Echis* spp., and *Naja* spp. specific equine F(ab)’_2_ antivenom formerly manufactured by Sanofi Pasteur, France) [16] and FitzSimons’s antivenomous serums (a discontinued polyvalent anti *Bitis* spp., *Hemachatus* spp., and *Naja* spp. equine antivenom) [4] were each used to treat one patient. The name of the antivenom was not recorded for seven patients, but the authors reported using a polyvalent antivenom [20,24,28–30]. No data was reported on adverse reactions for the other antivenoms.

Surgical interventions were performed on 15 (41%) patients [4,15,16,18,19,21,22,25,28–30]. Eleven (30%) patients developed necrosis at the bite site, occurring between 24 hours and 1 week after the bite [4,19,21,22,25,28,30]. A surgical debridement was performed on nine (11%) patients [4,15,16,19,21,28,30], and four (11%) patients had a digit amputation [22,25,28,30], with the amputation being preceded by a debridement in three cases [22,25,30]. A fasciotomy was performed on four patients for a suspected compartment syndrome [15,18,29,30]. No loss of function was described in surviving patients, and only one patient required skin grafting [19].

There were four (11%) fatal cases in our review [4,15,33]. Two of these patients did not receive antivenom and died from persistent hypotension and severe anaemia, resistant to IV fluids and the transfusion of blood products [4,33]. The remaining two were treated with SAIMR polyvalent antivenom; one patient died from repeated lower respiratory tract infections and overwhelming sepsis [15], while the second patient developed post operative complications (severe electrolyte imbalance and ventricular fibrillation) after a delayed amputation of his right leg for gangrene [4].

### Captive vs wild puff adder envenoming

**Fig 3:**
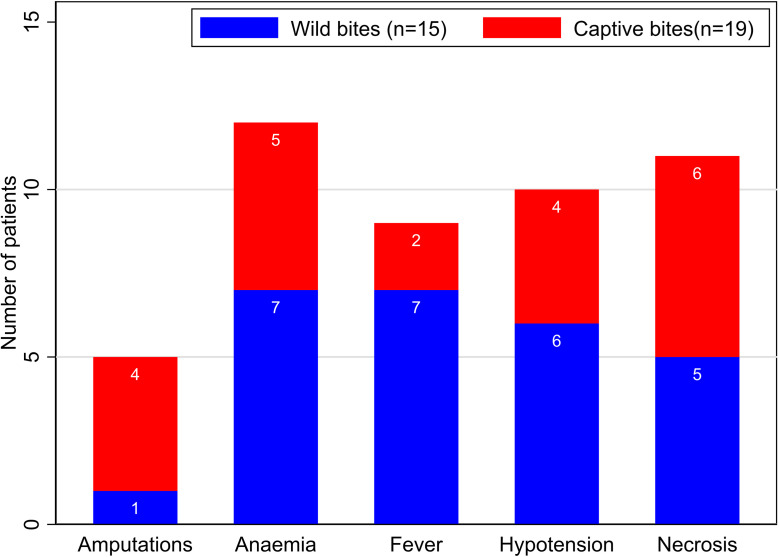
Clinical features and complications of puff adder bites in patients with wild and captive bites. *Some patients had more than one feature or complication. The origin of the snake was not reported in three cases.

We compared the major clinical features of envenoming following bites by wild (15 patients) or captive (19 patients) snakes to explore any patterns that can influence management and outcomes. Captive bites were more common on the digits (68%) [21,22,25,26,28–31,34], compared to wild bites in which the bite sites were more varied. Compared to patients with captive bites, patients with wild bites had a higher documented occurrence of hypotension (21% [25,27,33,34] vs 40% [4,16,19]), fever (11% [25,29] vs 47% [4,17,19]), and anaemia (26% [23,25,30,33,34] vs 47% [4,16,17,19]). The occurrence of bite site necrosis (32% [22,25,28,30] vs 33% [4,16,19]) was similar in both groups, while patients with captive bites had a higher occurrence of amputations (21% [22,25,28,30] vs 7% [4]). These differences were not statistically significant (p=0.35), and Fig 3 below compares the absolute number of patients with each of the above features or complications of puff adder bites.

### Laboratory features of puff adder envenoming reported in the literature

Serial laboratory measures were only reported in three patients to allow an assessment of evolution of laboratory features of envenoming, and these are presented in Table 3. There was a haemoglobin drop of 2.3g/dl [21], 4.5g/dl [34] and 4.6g/dl [33] in all three patients. All patients had at least one episode of thrombocytopenia, with levels as low as 34,000 cells per microliter in one patient [33]. Only one patient had a marked leucocytosis, with white blood cell count increasing from 6400 to 17500 cells per microliter [33]. Coagulation assays were mildly abnormal. The fibrinogen concentration remained normal in all three patients, while the prothrombin time/INR and APTT were normal in one patient [21], mildly raised in a second (max INR 1.38) [34], and more raised in the third (max INR 2.93) [33]. Serum electrolyte results were available for only one patient [33], and they had a severe hypokalaemia (2.5 millimoles per litre) on admission, and an acute kidney injury with a progressive increase in serum creatinine levels from 68.8 to 261.3 micromoles per litre. Serial creatine kinase levels were reported for two patients [33,34] and these increased progressively over time, ranging between 90 and 330 units per litre.

**Table 3:** Serial laboratory assays of published cases of puff adder envenoming.

Author	Timepoints	Haematology			Coagulation assays				Biochemistry				
		Hb	WBC	Plt	PT	INR	APTT	Fib	K+	Na+	Creat	Urea	CK
Bey et al [21]	Admission + 0hrs	15.4	10	151	13.4	1.3	31.6	235	–	–	–	–	–
	Early Admission + 6hrs	14.8	–	149	–	–	–	–	–	–	–	–	–
	Late Admission+74hrs	13.1	–	178	11.6	0.9	29.8	480	–	–	–	–	–
Wakasugi et al [34]	Admission + 0hrs	14.2	6.7	220	–	0.98	26	–	–	–	–	–	90
	Late Admission+24hrs	11.3	9.4	109	–	1.38	43	294	–	–	–	–	303
	Late Admission+48hrs	9.7	9.7	123	–	1.24	43	302	–	–	–	–	299
Husain et al [33]	Admission + 3hrs	16.5	6.4	101	17.6	1.28	38.5	–	2.5	140	68.8	3.2	191
	Early Admission + 9hrs	16.8	16.8	34	24.0	1.76	47.9	–	3.7	136	80.1	4.1	197
	Late Admission+18hrs	11.9	17.5	41	39.3	2.91	95.5	–	4.3	140	261.3	6.8	229

Reference values and abbreviations: Hemoglobin (Hb) 13.0–17.0 g/dL; White Blood Cells (WBC) 4.0–10.0 x 109/L; Platelet count (Plt) 150–410 x 109/L; Prothrombin Time (PT)11.6–14.9 seconds; Activated Partial Thromboplastin Time 30.3–46.5 seconds; International Normalised Ratio (INR);) 0.8–1.1; Fibrinogen concentration (Fib) 200–400mg/dl;Urea 2.1–7.1 mmol/L; Creatinine 63.6–110.5 umol/L; Sodium 136–145 mmol/L; Potassium 3.5–5.1 mmol/L; Creatinine Kinase 30–200U/L.

### Discussion

Despite being a medically important snake with a wide geographical distribution, puff adder envenoming has been rarely described in the published literature. Our review found only 37 cases that have been described since 1970 with sufficient detail to understand the local and systemic features of puff adder envenoming, with only three of these cases being reported with serial laboratory assays. We added this review of published cases to the clinical reports of 15 cases of puff adder envenoming from two hospitals in Kenya reported in this study.

### Geographic specificities

Puff adders are native to the African continent and the Middle East. In our literature review, 54% (20/37) of previous cases of envenoming occurred in sub-Saharan Africa. The profile of patients presenting with a puff adder bite on the continent was heterogenous, including farmers, school children, and herpetologists. From our case series, the most common formal occupation was herding, and a significant proportion of patients had no formal occupation. Bites occurring outside the African continent were always the result of individuals getting accidentally bitten while maintaining a captive snake, and were more likely to be published [21–23,25–31,33,34]. This over representation of captive bite limits our understanding of puff adder envenoming in the more common rural environment. These geographic differences were also evident from the most commonly bitten body part, which was the lower limb in 66% (23/35) of all puff adder bites that happened on the African continent (case series and literature review), and the upper limb in 82% (14/17) of puff adder bites occurring outside the African continent (literature review only) [13–15,18,20–31,33,34].

### Biting snake identification

Despite the broad geographic coverage of puff adders, very few cases of confirmed puff adder bites have been reported in the literature. This is likely due to challenges in identifying the biting species in the field. Definitive identification requires the use of immunological laboratory assays, or a herpetologist to assess a picture, or parts of the biting snake. This infrastructure and expertise are not always present in areas where the most snakebites occur. In a recent review, snakebite identification by immunoassay or a herpetologist was only used in 39 and 23 of 150 publications on snakebite, respectively [35]. Most publications used less objective methods such as verbal description by the patient or bystanders, and clinical features of envenoming. This is particularly relevant for puff adder envenoming because studies in Ethiopia [36], Kenya [37,38], Tanzania [39], Zimbabwe [3,40], and South Africa [41] have reported high burdens of envenoming by puff adder, representing up to 75% of snakebites in some studies. Unfortunately, the method of biting snake identification was not consistently reported. This is evident in our case series where the biting snake species was only objectively confirmed in two out of 15 cases. There still exists huge gaps in the diagnosis of snakebite envenoming and in the identification of the responsible biting species, and efforts to address these limitations would have the added benefit of reducing the time to treatment and improving patient outcomes.

### Local features

The most common local symptoms of puff adder envenoming were pain and swelling. Fang marks were a common feature after a bite, they were visible in all patients in our case series and were mentioned in 50% of studies in our review. Two recent reviews on dry bites did not find a case of a dry puff adder bite [42,43], but this may be substantially influenced by health seeking behaviours. Although the relevance of fang marks in the diagnosis of snakebite envenoming is debated, puff adders have large fangs, measuring 16–18 mm in length, and this muscular snake species can strike with force, likely increasing the chances of envenoming [1]. The occurrence of dry bites after bites by other medically important snake species, including members of the *Echis* and *Naja* genera, have been reported to range between 8 and 30% [44,45]. Nonetheless, as with all other cases of snakebite, patients presenting with a suspected puff adder bite, with or without fang marks, should be hospitalised and kept under close observation for at least 24 hours.

Local swelling and pain are well-described symptoms, and they are likely due to the effects of snake venom metalloproteinase (SVMP), phospholipases A_2_ (PLA_2_) and other cytotoxic venom toxins [46,47]. They cause local tissue damage by hydrolysing extracellular matrix components such as collagens, hyaluronic acid, and proteoglycans [47–49]. Most venom components are absorbed through the lymphatic system [50], and as with many other species, this likely explains the tender swollen lymph nodes proximal to the bite site. Other less well understood local symptoms include blister formation, necrosis, and erythema. The variability in manifestation between patients, and the specific mechanisms underlying these symptoms remain poorly understood.

### Systemic symptoms

In our case-series, haemorrhage was the most commonly reported systemic feature of puff adder envenoming, followed by coagulopathy and hypotension. From the review, hypotension, fever, and anaemia were the most commonly reported systemic features of puff adder envenoming. These three features—haemorrhage, coagulopathy, and hypotension, did occur independently but could also be linked. The haemorrhage from puff adder bites is said to most often be local, from the bite site, and can persist for up to 48 hours after the bite [16]. *Bitis arietans* venom also contains proteins and peptides that can impair platelet function [51–59] or directly damage platelets, resulting in thrombocytopenia [60–62]. A combination of platelet inactivation and depletion therefore likely contribute to the prolonged local haemorrhage observed in puff adder envenoming.

We also described cases of less clinically obvious haemorrhage in our review. Two patients had subcutaneous haemorrhage into the arm and lower back after a bite to the finger [33,34], and one patient developed a haematoma in the jaw after a bite to the foot [19]. These accounts are suggestive of more than perturbed platelet function. SVMPs and snake venom serine proteases (SVSPs), both of which are found in puff adder venom, can hydrolyse type I and IV collagen in the basement membranes of blood vessels (mostly capillaries), causing microvascular damage, increasing vascular permeability and leading to the extravasation of blood into subcutaneous or other concealed spaces [47,48,63]. An autopsy in one of the fatal cases of puff adder envenoming revealed haemorrhages in the walls of the small intestine and into the adventitia of the aorta [4]. The contribution of coagulopathy in these cases of concealed haemorrhage is uncertain, and a future robust assessment of coagulation parameters is needed for further clarification. *Bitis* spp. venom also contains SVSPs that act as thrombin-like enzymes and therefore also possess fibrinogenolytic activity and can cause coagulopathy [46,51,64]. This coagulopathy is less severe when compared to the venom induced consumptive coagulopathy (VICC) observed after envenoming by the related saw-scaled vipers (*Echis* spp.). The highest serially measured INR in our scoping review was 2.9 [33], much lower than the unrecordable INRs (i.e., >7) frequently seen with *Echis* spp. envenoming [65]. We did not find any evidence of complete fibrinogen consumption, as when measured, fibrinogen levels were normal in the two studies that reported serial tests, suggesting a more prominent role of vessel wall degradation and platelet dysfunction, than consumption of coagulation factors and ensuing coagulopathy. However, these assays were performed in a very limited number of patients and results should be interpreted with caution. There are five snake species that can cause haemotoxic envenoming in Kenya, two vipers (*Bitis arietans*, and *Echis pyramidum*) and three colubrids (*Dyspholidus typhus*, *Thelotornis mossambicanus* and *Thelotornis usambaricus*). The colubrids are tree dwelling snakes, they are category 2 medically important snakes, making them unlikely to be encountered and unlikely to be mistake for any of the vipers. *Echis pyramidum* does not occur naturally in Kitui county and is only found in the Northern parts of Baringo county where there may have been a small range of overlap with *Bitis arietans*, leaving a low residual probability that the biting snake species in Baringo county were not puff adders, but this risk is minimal.

Hypotension in patients with puff adder envenoming can be a direct action of venom toxins, or an indirect consequence of haemorrhage and limb swelling. *Bitis* spp. venom contains large amounts of adenosine [66] and also bradykinin potentiating peptides [67] and kinin-releasing SVSPs [68] that cause the release of bradykinin and other vasoactive mediators which inhibit angiotensin converting enzyme and lead to haemodynamic disturbance [64,66]. There is also evidence that SVMPs in puff adder venoms can directly generate the vasodilator angiotensin 1–7 [69]. Another known component of puff adder venom, vascular endothelial growth factor [46], can also be a cause of hypotension. Hypotension can also result from the reduction in circulating blood volume following microvascular damage and extravasation of blood from the capillaries, or extravasation of fluid into swollen limbs or body parts. The acute nature of hypotension from puff adder envenoming potentially carries a high risk of fatality from shock [4], making it an important feature of envenoming. This should be actively sought out and managed, because a timely fluid bolus and appropriate antivenom can quickly reverse hypotension in patients with puff adder envenoming and improve their prognosis [4,25,27,34]. Some authors recommend serial and close monitoring of the blood pressure and pulse in patients with puff adder envenoming for 48 hours [4].

Fever was another common systemic feature in our review, often accompanied by local signs of inflammation such as swelling and pain but also occurring without signs of wound infection or necrosis. *Bitis arietans* venom has been shown in-vivo to strongly activate the inflammatory process, with the induction and production of inflammatory mediators—cytokines, chemokines and eicosanoids [68,70]. This marked production of potent endogenous pyrogens such as IL-1β, IL-6 and TNF-α, can interact directly with the anterior hypothalamus, through a hierarchy of neural structures, and raise the temperature setpoint, causing fever [71,72]. They also activate the endothelium, inducing vasodilation and increased vascular permeability, and activate hepatocytes, inducing the production of acute phase proteins which amplify the inflammatory process [68,73].

While a discussion of treatment options and outcomes are beyond the scope of this paper, we would like to highlight the use of fasciotomies in four out of six patients with clinical suspicion of compartment syndrome reported in the literature. The role of surgery in the management of puff adder envenoming is uncertain and compartment syndrome is rare. The extreme swelling after puff adder envenoming means that clinical signs such as pulselessness, pain, paraesthesia, and cold extremities are common and can cause overdiagnosis of compartment syndrome; these should always be accompanied by measurements of intra-compartmental pressure, or by ultrasound imaging to rule out vascular compromise. The two patients who were managed conservatively made a full recovery, suggesting timely administration of antivenom, alongside supportive treatment such as intravenous fluids may be sufficient.

Limitations in the scoping review methodology, especially our limited search of grey literature sources, may have contributed to the under reporting of puff adder bites in African countries, and reduced the validity of the features of envenoming reported in this study. Publication and observer bias may also skew our understanding of the pathophysiology of puff adder envenoming towards envenoming by captive snakes over wild snakes. Limitations in the accurate identification of the biting snake and in serial laboratory assays hinder progress in forming a global understanding of the pathophysiology of puff adder envenoming. This was most evident in our case series where only two of the biting snakes were objectively identified as puff adders, limiting the accuracy of our description of clinical features of puff adder envenoming. Nonetheless, by combining data on 52 cases of puff adder envenoming we enabled the synthesis of common local and systemic signs, revealed key features of envenoming that require different therapeutic approaches, and highlighted some differences between envenoming by captive and wild puff adders.

## Conclusion

The puff adder is a medically important snake in Africa and the middle East, yet there are still gaps in the understanding of envenoming after a bite. We demonstrate that the most common local symptoms of puff adder envenoming are pain and swelling, while fang marks are typically easily visible after a bite. Systemic features include haemorrhage, coagulopathy, and hypotension, which may have a shared pathophysiology, and require timely recognition and correction to avoid fatal outcomes. Fever is a less commonly reported but frequent feature of puff adder envenoming, and it can be an early indicator of envenoming in patients with subtle local or systemic signs. Definitive identification of the biting snake, and clinical research with serial laboratory assays, is crucial to further understand the pathophysiology of envenoming by this medially important snake species.

## Supporting information

S1 Checklist PRISMA checklist for scoping reviews. (DOCX)

S2 Checklist PLOS inclusivity in global research checklist. (DOCX)

S1 Table Details of clinical features of patients included in case series. (DOCX)
