# Supplementary material for: Clinical features of puff adder envenoming: case series of Bitis arietans snakebites in Kenya and a scoping review of the literature
Source: PLoS Negl Trop Dis. 2025 Feb 10;19(2):e0012845. doi: 10.1371/journal.pntd.0012845 (PMC11828387; doi:10.1371/journal.pntd.0012845)
Supplement: S1 Table — (DOCX) [file pntd.0012845.s003.docx]

Supplementary Table 1 – Details of clinical features of patients included in case series

| **ID No** | | **Age**  **(yrs)** | **Gender** | | | **Hosp delay (hrs)** | | | **Bite location** | | | **Identification of biting snake** | | | **Antivenom indication** | | | | | **Antivenom within 1 hr of arrival** | | **Patient outcome** | | |
| --- | --- | --- | --- | --- | --- | --- | --- | --- | --- | --- | --- | --- | --- | --- | --- | --- | --- | --- | --- | --- | --- | --- | --- | --- |
| 01 | | 40 | Female | | | 9.3 | | | Ankle/knee | | | Picture of dead snake | | | Rapid progressive swelling (RPS) + Swelling > 1/2 limb | | | | | Yes | | Discharged home after 3 days | | |
| 02 | | 24 | Female | | | 10.6 | | | Hand/foot | | | Clinical symptoms + Picture chart | | | RPS + Swelling to fingers/toes + Across 2 joints | | | | | Yes | | Discharged home after 2 days | | |
| 03 | | 12 | Female | | | 11.9 | | | Neck/head | | | Clinical symptoms + Picture chart | | | Spontaneous bleeding + RPS + Swelling > 1/2 limb | | | | | Yes | | Discharged home after 3 days | | |
| 04 | | 21 | Male | | | 7 | | | Toes/foot | | | Clinical symptoms + Picture chart | | | RPS + Swelling to fingers/toes + Across 2 joints | | | | | Yes | | Discharged home after 1 day | | |
| 05 | | 99 | Male | | | 5.3 | | | Toes/foot | | | Clinical symptoms + Picture chart | | | RPS + Swelling across 2 joints + > 1/2 limb | | | | | Yes | | Discharged home after 2 days | | |
| 06 | | 14 | Female | | | 13 | | | Arm/shoulder | | | Clinical symptoms + Picture chart (Snake killed) | | | RPS + Swelling across 2 joints + > 1/2 limb | | | | | Yes | | Discharged home  After 2 days | | |
| 07 | | 78 | Male | | | 11.3 | | | Wrist/elbow | | | Clinical symptoms + Picture chart | | | RPS + Swelling to fingers/toes | | | | | Yes | | Discharged home  After 2 days | | |
| 08 | | 28 | Male | | | 6.7 | | | Toes/foot | | | Clinical symptoms + Picture chart | | | RPS + Swelling to fingers/toes | | | | | Yes | | Discharged home after 3 days | | |
| 09 | | 23 | Female | | | 7 | | | Ankle/knee | | | Clinical symptoms + Picture chart | | | RPS + Swelling to fingers/toes + Across 2 joints + > 1/2 limb | | | | | Yes | | Discharged home after 1 day | | |
| 10 | | 20 | Male | | | 0.5 | | | Toes/foot | | | Clinical symptoms + Picture chart | | | RPS | | | | | Yes | | Discharged home after 3 days | | |
| 11 | | 16 | Male | | | 2 | | | Ankle/knee | | | Clinical symptoms + Picture chart | | | RPS + Swelling across 2 joints + > 1/2 limb | | | | | Yes | | Discharged home after 3 days | | |
| 12 | | 19 | Male | | | 1.5 | | | Toes/foot | | | Clinical symptoms + Picture chart | | | RPS + Swelling > 1/2 limb | | | | | Yes | | Discharged home after 3 | | |
| 13 | | 15 | Female | | | 0 | | | Toes/foot | | | Brought dead snake | | | RPS + Swelling > 1/2 limb | | | | | Yes | | Discharged home after 5 days | | |
| 14 | | 35 | Female | | | 7.3 | | | Ankle/knee | | | Clinical symptoms + Picture chart | | | RPS + Swelling > 1/2 limb | | | | | Yes | | Discharged home after 4 days | | |
| 15 | | 26 | Male | | | 0.8 | | | Ankle/knee | | | Clinical symptoms + Picture chart | | | RPS + Swelling > 1/2 limb | | | | | Yes | | Discharged home after 5 days | | |
| **Systemic features of hemotoxic envenoming** | | | | | | | | | | | | | | | | | | | | | | | | |
| **ID No** | **20 minute whole blood clotting test (20 WBCT)** | | | | | | | | | | | | **Prolonged bleeding from venepuncture site** | | | | | **Gingival haemorrhage** | | | | | | **GIT haemorrhage** |
|  | 0hr | | | 6hr | | | 12hr | | | 24hr | | | 0hr | 6hr | | 12hr | 24hr | 0hr | 6hr | | 12hr | | 24hr | 0 - 24hr |
| 01 | Abnormal | | | Normal | | | Normal | | | Normal | | | Yes | No | | No | No | Yes | Yes | | No | | No | No |
| 02 | Normal | | | Normal | | | Normal | | | Normal | | | No | No | | No | No | No | No | | No | | No | No |
| 03 | Abnormal | | | Abnormal | | | Normal | | | Abnormal | | | Yes | No | | No | No | Yes | Yes | | No | | No | No |
| 04 | Normal | | | Normal | | | Normal | | | Normal | | | No | No | | No | No | No | No | | No | | No | No |
| 05 | Normal | | | Normal | | | Normal | | | Normal | | | No | No | | No | No | No | No | | No | | No | No |
| 06 | Normal | | | Normal | | | Normal | | | Normal | | | No | No | | No | No | No | No | | No | | No | No |
| 07 | Normal | | | Normal | | | Normal | | | Normal | | | No | No | | No | No | No | No | | No | | No | No |
| 08 | Normal | | | Normal | | | Normal | | | Normal | | | No | No | | No | No | No | No | | No | | No | No |
| 09 | Normal | | | Normal | | | Normal | | | Normal | | | No | No | | No | No | No | No | | No | | No | No |
| 10 | Normal | | | Normal | | | Normal | | | Normal | | | No | No | | No | No | No | No | | No | | No | No |
| 11 | Normal | | | Normal | | | Normal | | | Normal | | | No | No | | No | No | No | No | | No | | No | No |
| 12 | Normal | | | Normal | | | Normal | | | Normal | | | No | No | | No | No | No | No | | No | | No | No |
| 13 | Normal | | | Normal | | | Normal | | | Normal | | | No | No | | No | No | No | No | | No | | No | No |
| 14 | Normal | | | Normal | | | Normal | | | Normal | | | No | No | | No | No | No | No | | No | | No | No |
| 15 | Normal | | | Normal | | | Normal | | | Normal | | | No | No | | No | No | No | No | | No | | No | No |
| **Other systemic features of envenoming** | | | | | | | | | | | | | | | | | | | | | | | | |
| **ID No** | | **Tachycardia** | | | | | | | | | | | **Hypotension** | | | | | **Fever** | | | | | | |
|  |  | 0hr | | | 6hr | | | 12hr | | | 24hr | | 0hr | 6hr | | 12hr | 24hr | 0hr | 6hr | | 12hr | | 24hr | |
| 01 | | Yes | | | No | | | No | | | No | | No | No | | No | No | No | No | | No | | No | |
| 02 | | No | | | No | | | No | | | No | | No | No | | No | No | No | No | | No | | No | |
| 03 | | Yes | | | Yes | | | No | | | No | | Yes | Yes | | No | No | Yes | No | | No | | No | |
| 04 | | No | | | No | | | No | | | No | | No | No | | No | No | No | No | | No | | No | |
| 05 | | No | | | No | | | No | | | No | | No | No | | No | No | No | No | | No | | No | |
| 06 | | No | | | No | | | No | | | No | | No | No | | No | No | Yes | No | | No | | Yes | |
| 07 | | No | | | No | | | No | | | No | | No | No | | No | No | No | No | | No | | Yes | |
| 08 | | No | | | No | | | No | | | No | | No | No | | No | No | No | No | | No | | No | |
| 09 | | No | | | No | | | No | | | No | | No | No | | No | No | No | No | | Yes | | No | |
| 10 | | No | | | No | | | No | | | No | | No | No | | No | No | No | No | | No | | No | |
| 11 | | No | | | No | | | No | | | No | | No | No | | No | No | No | No | | No | | No | |
| 12 | | No | | | No | | | No | | | No | | No | No | | No | No | No | No | | No | | No | |
| 13 | | Yes | | | No | | | No | | | No | | No | No | | No | No | No | No | | No | | No | |
| 14 | | No | | | Yes | | | No | | | No | | No | No | | No | No | No | Yes | | No | | No | |
| 15 | | No | | | No | | | No | | | No | | No | No | | No | No | No | No | | No | | No | |
| **Local features of envenoming** | | | | | | | | | | | | | | | | | | | | | | | | |
| **ID No** | | **Swelling** | | | | | | | | | | | **Bleeding from bite site** | | | | | **Pain** | | | | | | |
|  |  | 0hr | | | 6hr | | | 12hr | | | 24hr | | 0hr | 6hr | | 12hr | 24hr | 0hr | 6hr | | 12hr | | 24hr | |
| 01 | | Yes | | | Yes | | | Yes | | | Yes | | No | No | | No | No | Severe | Severe | | Moderate | | Mild | |
| 02 | | Yes | | | Yes | | | Yes | | | Yes | | Yes | No | | No | No | Very severe | Severe | | Severe | | Moderate | |
| 03 | | Yes | | | Yes | | | Yes | | | Yes | | Yes | Yes | | No | No | Worst possible | Very severe | | Severe | | Severe | |
| 04 | | Yes | | | Yes | | | Yes | | | Yes | | No | No | | No | No | Severe | Moderate | | Mild | | Mild | |
| 05 | | Yes | | | Yes | | | Yes | | | Yes | | No | No | | No | No | Severe | Severe | | Moderate | | Mild | |
| 06 | | Yes | | | Yes | | | Yes | | | . | | No | No | | No | No | Moderate | Moderate | | Moderate | | . | |
| 07 | | Yes | | | Yes | | | Yes | | | . | | No | No | | No | No | Mild | Mild | | Mild | | . | |
| 08 | | Yes | | | Yes | | | No | | | No | | No | No | | No | No | Mild | Mild | | Mild | | Mild | |
| 09 | | Yes | | | Yes | | | Yes | | | Yes | | Yes | Yes | | No | No | Very severe | Severe | | Severe | | Mild | |
| 10 | | Yes | | | Yes | | | Yes | | | Yes | | No | No | | No | No | Very severe | Severe | | Moderate | | Moderate | |
| 11 | | Yes | | | Yes | | | Yes | | | Yes | | Yes | No | | No | No | Severe | Severe | | Moderate | | Moderate | |
| 12 | | Yes | | | Yes | | | Yes | | | Yes | | Yes | No | | No | No | Severe | Severe | | Moderate | | Mild | |
| 13 | | Yes | | | Yes | | | Yes | | | Yes | | Yes | Yes | | No | No | Very severe | Severe | | Severe | | Mild | |
| 14 | | Yes | | | Yes | | | Yes | | | Yes | | Yes | No | | No | No | Very severe | Severe | | Severe | | Moderate | |
| 15 | | Yes | | | Yes | | | Yes | | | Yes | | Yes | No | | No | No | Severe | Severe | | Severe | | Moderate | |
